# Investigation on Fuel Quality and Combustion Characteristics of Blended Fuel (Biomass and Lignite) Derived from Low-Temperature Co-Upgradation

**DOI:** 10.3390/molecules30163435

**Published:** 2025-08-20

**Authors:** Ning Liu, Bohao Bai, Xu Yang, Zhuozhi Wang, Boxiong Shen

**Affiliations:** School of Chemical Engineering and Technology, Hebei University of Technology, Tianjin 300401, China; 202331505006@stu.hebut.edu.cn (N.L.); 222002@stu.hebut.edu.cn (B.B.); 202511501026@stu.hebut.edu.cn (X.Y.)

**Keywords:** biomass, lignite, co-upgradation, fuel quality, hydrophobicity, combustion characteristics

## Abstract

Co-combustion is regarded as an effective means for high-efficiency utilization of low-quality fuels. However, low-quality fuel has problems such as low energy density and high water content. The fuel quality and blending performance can be further optimized by the pretreatment of low-quality fuel, for example, calorific value, hydrophobicity, and NO conversion rate. Based on the idea of co-upgradation, this study systematically investigates the effects of integrated upgrading on fuel quality and hydrophobicity under different conditions. In this study, lignite and wheat straw were selected as research objects. The co-upgrading experiments of wheat straw and lignite were conducted at reaction temperatures of 170 °C, 220 °C, and 270 °C in flue gas and air atmospheres with biomass blending ratios of 0%, 25%, 50%, 75%, and 100%. SEM (scanning electron microscopy) and nitrogen (N_2_) adsorption analyses showed that under low-temperature and low-oxygen conditions, organic components from biomass pyrolysis migrated in situ to cover the surface of lignite, resulting in a gradual smoothing of the fuel surface and a decrease in the specific surface area. Meanwhile, water reabsorption experiments and contact angle measurements showed that the equilibrium water holding capacity and water absorption capacity of the lifted fuels was weakened, and hydrophobicity was enhanced. Combustion kinetic parameters and pollutant release characteristics were investigated by thermogravimetric analysis (TGA) and isothermal combustion tests. It was found that co-upgradation could effectively reduce the reaction activation energy and NO conversion rate. Characterized by Raman spectroscopy (Raman) and X-ray photoelectron spectroscopy (XPS), in situ migration of organic components affected combustion reactivity by modulating changes in N-containing product precursors. The results showed that the extracted fuel with a 75% biomass blending ratio in the flue gas atmosphere exhibited the best overall performance at 220 °C, with optimal calorific value, combustion reactivity, and hydrophobicity. These findings may provide important theoretical foundations and practical guidance for the optimization of industrial-scale upgrading processes of low-quality fuels.

## 1. Introduction

Energy is the driving force behind socioeconomic development. Coal is the primary energy source for combustion power generation and heating in traditional Chinese power plants [1]. Due to factors such as excessive carbon emissions, worsening environmental pollution, and insufficient fossil fuel supplies, biomass energy, which is abundant and carbon-neutral, is considered an economically rational alternative to coal. Lignite, the lowest rank of coal, has a global proven reserve of approximately 80 billion tons, accounting for 45% of the total coal reserves [2]. In China, the proven lignite reserves are about 31.94 billion tons, representing 16.2% of the country’s total coal reserves [2]. Due to its low cost, which is only one-third that of bituminous coal, lignite has attracted global attention. However, lignite is characterized by a high moisture content (25–65 wt.%), low energy density, and a strong tendency towards spontaneous combustion, all of which severely restrict its clean and efficient utilization as well as safe storage and transportation [3]. Biomass resources are abundant and low in price, with an annual global production exceeding 10 billion tons [4]. Nevertheless, biomass is also characterized by high moisture content, low energy density, and poor thermal stability, making it difficult to utilize directly. Therefore, the integrated treatment of these abundant low-quality fuel resources, which are so rich in global reserves, to achieve their resourceful and sustainable utilization is crucial for realizing the clean, efficient, and low-carbon utilization of coal and promoting renewable energy as the main source of future energy supply increments.

In previous studies, co-combustion of coal with biomass has been identified as one of the effective pathways for the utilization of coal-based fuels and biomass. Despite the lower heating value of biomass, the release of volatile matter during the initial combustion phase helps to increase the overall flame temperature and enhance the reactivity of the fuel [5]. Guo et al. [6] granulated wheat straw, sawdust, and willow catkins into biomass pellets and used thermogravimetric analysis to study the combustion process and reactivity of these biomass pellets when co-combusted with lignite. The heat released from the combustion of biomass pellets accelerated the pyrolysis and combustion reactions of coal, promoting the release of volatile matter and the oxidation of char. As the biomass pellet blending ratio increased from 0% to 70%, the burnout temperature of the blended fuel decreased from 900 °C to 730 °C, and the comprehensive combustion characteristic index increased from 0.33 × 10^−7^ to 3.78 × 10^−7^. In addition, for co-combustion power plant boilers, the co-combustion of biomass with coal not only significantly improves fuel utilization efficiency but also greatly reduces costs. Compared with biomass boilers, the addition of coal-based fuels can relatively lower the alkali metal content, thereby reducing the corrosion of boiler equipment [7]. When biomass samples enter the boiler, the initial release and rapid combustion of volatile matter create a hypoxia atmosphere, which inhibits the formation of NO and SO_2_. Liao et al. [8] and Dong et al. [9] demonstrated that coal–biomass blends significantly reduce pollutant emissions while enhancing combustion reactivity and burnout rates. The co-combustion of lignite and biomass can be regarded as an effective approach for the clean and efficient utilization of these two low-quality fuels. However, previous studies have predominantly focused on the direct co-combustion of lignite and biomass without pre-combustion blending and pre-treatment, resulting in significant differences in the properties of biomass and lignite during the initial combustion phase. Therefore, pre-combustion upgrading and modification of lignite and biomass are considered effective means to produce a high-quality blended fuel.

Numerous upgrading technologies have been developed for individual pretreatment of lignite or biomass. For lignite, conventional thermal drying reduces moisture content and spontaneous combustion risk. However, its high energy intensity and inability to confer persistent hydrophobicity result in significant water re-adsorption [10,11,12]. Simultaneous achievement of deep dehydration and durable hydrophobicity remains challenging: severe drying induces pore enlargement that elevates water uptake capacity, while high-temperature modification degrades coal’s reactive macromolecular structures [13,14]. Studies demonstrate that upgrading the treatment of lignite effectively enhances its fuel properties, including reduced moisture re-adsorption, elevated calorific value, and optimized combustion performance [1,15,16]. Currently, petroleum coke and asphalt are commonly used as additives to improve the surface structure of lignite after drying, which can effectively reduce moisture re-adsorption [17,18]. The volatile organic aromatic hydrocarbons released from asphalt or petroleum coke can penetrate and adhere to the pores of lignite particles, forming a coating that reduces the contact between moisture and active surfaces at ambient temperature. At elevated temperatures, these organic components are rapidly released and participate in combustion, thereby increasing the combustible components and heating value of lignite. However, these materials are costly and require high-temperature treatment, and using them as continuous, consumable modification additives inevitably leads to a significant increase in the upgrading cost of lignite. For biomass, low-temperature thermal upgrading is typically a necessary step to optimize fuel quality [19]. During low-temperature pyrolysis, moisture and light volatile components are removed from biomass, including benzene, toluene, styrene, phenol, and naphthalene. In the pyrolysis products, the energy density of the solid carbon char is significantly increased, and its hydrophobicity is improved; the volatile matter tar can be cracked to obtain water-resistant organic matter [20,21].

Based on the aforementioned studies, the co-combustion of biomass and lignite is an effective method for reducing pollutant emissions and improving fuel combustion reactivity. Both lignite and biomass are characterized by low heating values, low density, and high moisture content, requiring upgrading processes that simultaneously achieve low energy consumption, enhanced hydrophobicity, and protection of active structures. The polycyclic aromatic hydrocarbons generated from the low-temperature pyrolysis of biomass can be loaded into the pores of lignite particles, effectively improving the moisture re-adsorption capacity of dried lignite compared to asphalt or petroleum coke.

Therefore, this study proposes the co-upgrading of lignite and biomass under low-temperature conditions to prepare a lignite–biomass blended fuel. Co-upgradation denotes a process wherein lignite and biomass are blended at a fixed ratio, then undergo upgrading under controlled atmosphere and temperature conditions. To determine the impact of co-preprocessing and co-upgrading conditions on the characteristics of upgraded lignite–biomass fuels, this study conducted low-temperature co-upgrading experiments under various conditions, including different atmospheres (flue gas atmosphere and air atmosphere), temperatures (170 °C, 220 °C, 270 °C), and blending ratios (0%, 25%, 50%, 75%, 100%). The compositional changes of the low-temperature co-upgraded fuels were determined through elemental and proximate analyses. Subsequently, thermogravimetric analysis was employed to assess their combustion characteristics. The evolution of nitrogen speciation on the surface of samples under different co-upgrading conditions was investigated using Raman spectroscopy (Raman) and X-ray photoelectron spectroscopy (XPS). Additionally, the pore structure characteristics of the upgraded samples during low-temperature co-upgradation under various conditions were analyzed using nitrogen adsorption methods. The effects of low-temperature co-upgrading conditions on the hydrophobic properties of the upgraded fuels were explored through moisture re-adsorption experiments and contact angle measurements. This work elucidates the feasibility and mechanism of enhancing blended fuel hydrophobicity through volatiles released during in situ biomass pyrolysis under co-upgrading conditions. It further quantifies the synergistic effects of multi-parameter interactions on combustion performance through systematic experimental data. The co-upgrading of coal and biomass is of significant importance for the global management of large volumes of solid waste [9].

## 2. Results and Discussion

### 2.1. Analysis of Raw Material Characteristics Before and After Co-Upgradation

#### 2.1.1. Elemental and Industrial Analysis

**Figure 1:**
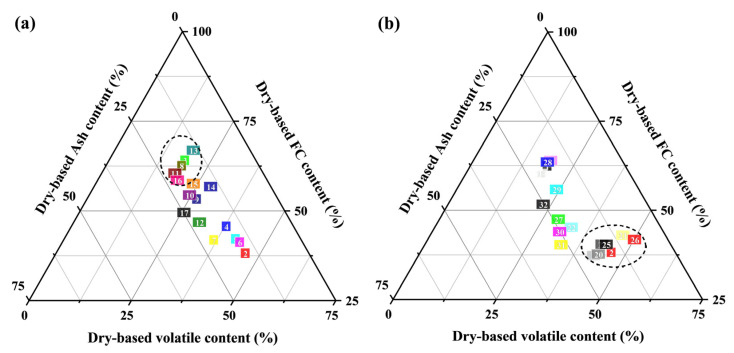
The distribution of three main components in dry-based samples: (**a**) flue gas atmosphere combined upgrading samples, (**b**) air atmosphere combined upgrading samples. The dotted circles indicate samples with relatively close ash content, volatile content and FC content.

As shown in Table 1, the calorific value of co-upgraded fuels under flue gas conditions exhibits significant improvement compared to raw lignite (R-L) and raw straw (R-S), while a decrease is observed for fuels treated in air. This pronounced reduction stems from combustible matter loss via intensive oxidation. After 220 °C flue gas pretreatment, the upgraded lignite achieved a calorific value of 30.19 MJ/kg—exceeding the 25.53 MJ·kg^−1^ reported for conventional Xiaolongtan lignite by Guo et al. [6] by 5.00 MJ·kg^−1^. Furthermore, the SLF-75-T220-F sample with high-biomass-ratio substitution exhibited a calorific value of 26.63 MJ/kg—representing an increase of approximately 1.20 MJ/kg over raw Xiaolongtan lignite and approaching its inherent calorific value. Figure 1 illustrates the distribution of key components—volatile matter, fixed carbon, and ash—in raw and co-upgraded samples. After co-upgradation, the fixed carbon content under flue gas conditions is higher than that under air conditions. Zhao et al. [22] reported that dewatering pretreatment increases the relative fixed-carbon content of upgraded coals by approximately 4%. Consistent with this trend, the fixed-carbon content of sample SLF-0-T220-F rose from 58.20% in R-L to 60.21%. Oxygen molecules in air and flue gas participate in low-temperature pyrolysis reactions during the drying process, altering the distribution of volatile matter and fixed carbon in modified fuels. Due to the higher oxygen content in air compared to flue gas, oxidative reactions are more intense in air, leading to substantial consumption of reactive combustible components (especially volatile matter), resulting in the loss of combustibles and a reduced calorific value. In contrast, the oxygen-deficient flue gas atmosphere suppresses oxidation, preserving fixed carbon.

Studies indicate that a bitumen coating in coal–oil slurry dehydration processes increases the calorific value of low-rank coal by approximately 0.5% [15]. However, as a consumable additive, bitumen significantly raises production costs. To address this limitation, we propose high-ratio biomass blending as an alternative pathway through a novel co-upgrading strategy. Under high biomass blending ratios, the calorific value of co-upgraded fuels initially increases and then decreases with rising pretreatment temperatures. For instance, the SLF-75-T220-F sample exhibits a calorific value of 26.63 MJ/kg, surpassing R-L (23.65 MJ/kg) by 2.98 MJ/kg and R-S (17.40 MJ/kg) by 9.23 MJ/kg. Notably, while the fixed carbon content of SLF-75-T220-F is lower than that of R-L, its calorific value increases. This phenomenon may arise from enhanced carbon retention in wheat straw-derived char at lower pyrolysis temperatures, which diminishes as the temperature escalates. Elevated temperatures during flue gas drying likely promote carbon release from straw, reducing the fixed carbon content. Tar, a combustible byproduct, contributes to the calorific value during combustion [23]. At lower temperatures, slow biomass pyrolysis limits volatile release due to insufficient thermal energy for complete macromolecular decomposition, hindering tar liberation. As the drying temperature gradually increases, the chemical bonds within the biomass begin to break, generating a large number of free radicals. These reactive intermediates recombine or interact with other molecules, forming larger aromatic compounds that condense into light tar fractions upon cooling. These tars deposit on lignite surfaces, filling pore structures and enhancing fuel energy density. This process explains the calorific value improvement despite reduced fixed carbon, as tar-derived hydrocarbons compensate for carbon loss. These findings suggest that under flue gas conditions, higher temperatures coupled with high biomass blending ratios synergistically improve fuel quality, approaching that of dried lignite. The modified fuel is cheap and easy to obtain, and its quality is close to that of dry lignite, providing a new idea for the large-scale utilization of biomass as an alternative fuel.

#### 2.1.2. SEM

**Figure 2:**
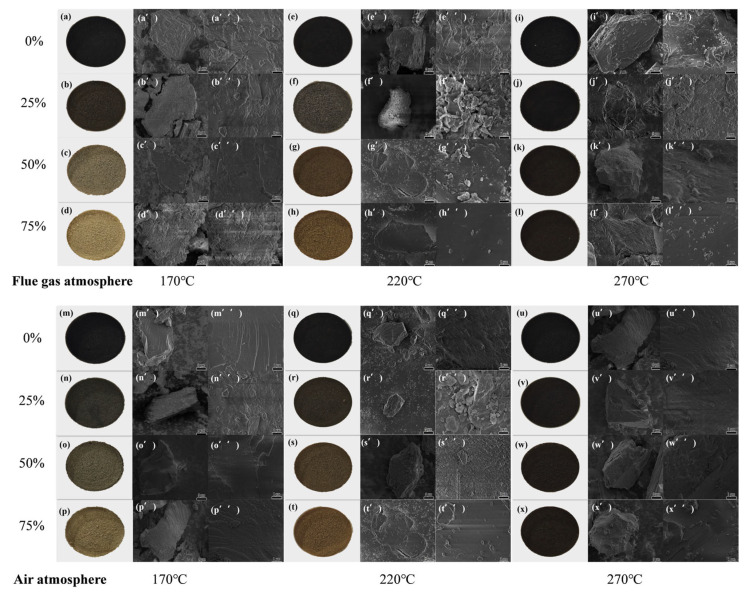
(**a**–**x**) Images of jointly upgraded samples; (**a′**–**x′**) 5000 times SEM images of jointly upgraded samples; (**a″**–**x″**) 20,000 times SEM images of jointly upgraded samples.

**Figure 3:**
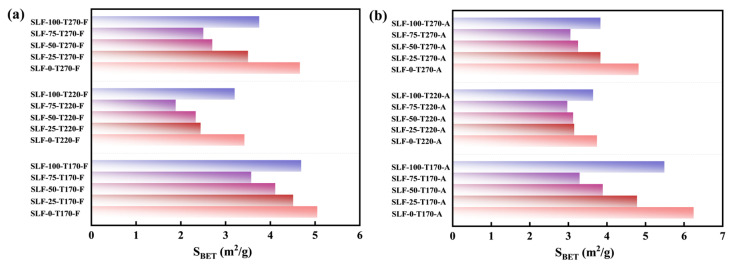
BET area distribution of the co-upgraded samples: (**a**) flue gas atmosphere; (**b**) air atmosphere.

Figure 2 clearly illustrates the effects of reaction temperature, wheat straw blending ratio, and atmosphere on the surface morphology of lignite–wheat straw blended fuels. As shown in Figure 2a–x, under lower pretreatment temperatures, SLF fuels undergo significant color transitions. Increasing the wheat straw blending ratio shifts the fuel color from its original state to brown tones, likely due to moisture removal and tar deposition on the surface. At 270 °C, all samples turn black regardless of blending ratio, indicating biomass carbonization into biochar [24]. Further analysis of Figure 2a′–x′ reveals that reaction temperature dominates surface structural evolution. Figure 2a″,e″ demonstrate that as the temperature increases to 220 °C, lignite particles exhibit smoothed surfaces with emerging cracks and fine particulate matter. This alteration may originate from the dehydration phase, during which the contraction forces acting on the particle surfaces lead to the collapse of certain pore structures. Consequently, both the number of mesopores and the specific surface area are reduced. According to IUPAC classification, coal pores are categorized into macropores, mesopores, and micropores, with the specific surface area (S_BET_) primarily governed by mesopores and micropores. Figure 3a shows that SLF-0-T220-F exhibits an S_BET_ of 3.43 m^2^/g, reduced by 1.63 m^2^/g compared to SLF-0-T170-F (5.06 m^2^/g), consistent with the correlation between higher S_BET_ and moisture retention in low-rank coals [25]. At 270 °C (Figure 3a), S_BET_ increases to 4.67 m^2^/g for SLF-0-T270-F, accompanied by intensified surface cracking. BET analysis suggests that thermal shrinkage from moisture evaporation collapses macropores into mesopores and micropores, restructuring the pore hierarchy.

Meanwhile, the S_BET_ of the jointly refined fuel SLF was smaller than that of the separately upgraded SLF-0-T(170~270)-(A/F), and the surface of the SLF-75-T220-F particles became smoother and flatter, with a minimum specific surface area of 1.89 m^2^/g. This was due to the fact that the roughness of the surface of the coal particles during the upgraded and dewatering process allowed the biomass pyrolysis tars to penetrate deeper into the pore interior, and the water originally occupying the fine pores was replaced by pyrolysis tar and its cracked aromatic components, which covered part of the pores. This change in the lignite surface is consistent with the results of Zhang et al.’s study on thermal modification of lignite with the addition of bitumen [17]. The temperature was further increased to 270 °C, and the surface of the modified fuel became rough instead. This is because the loss of volatile matter in the biomass due to the increase in temperature limits the effective formation and attachment of tar, thus reducing the degree of tar attachment on the surface of the SLF fuel. From Figure 3a,b, the optimum working conditions in both the flue gas atmosphere and air atmosphere are SLF-75-T220, and the S_BET_ (1.89 m^2^/g) of the co-extracted fuels obtained in the flue gas atmosphere is less than that of the extracted fuels obtained in the air atmosphere (2.98 m^2^/g). In the air atmosphere, the presence of oxygen leads to a more complete release of volatile components, which helps to open blocked pores and generate new micropores, thus increasing the specific surface area. In the flue gas atmosphere, the release of volatiles is limited by anoxic or low-oxygen conditions, which leads to the pyrolysis of volatiles into tars that block the pores and thus reduce the specific surface area. Based on the analysis of the surface morphology and pore structure characteristics of the particles, we can draw the following conclusions: at low temperatures, the surface of the fuel is gradually smoothed, and the specific surface area decreases with the increase in temperature. With further increases in temperature, the cracks on the surface are expanded, resulting in an increase in the specific surface area. In the flue gas atmosphere, low-oxygen conditions limit the release of volatile matter, causing the tar to clog the pores, thus reducing the specific surface area; in contrast, in the air atmosphere, high-oxygen conditions promote the opening of pores, thus increasing the specific surface area. The blending ratio affects the generation and attachment of tars, further altering the pore structure. The upgraded samples pretreated with 75% wheat straw doped in the flue gas atmosphere (6% O_2_ + 10% CO_2_ + 10% H_2_O + 74% Ar) exhibited a smaller specific surface area as well as smoother surfaces at higher temperatures (220 °C) than that at the low-temperature region.

### 2.2. Hydrophilicity Analysis in Co-Upgraded Raw Materials

#### 2.2.1. Contact Angle Characterization

**Figure 4:**
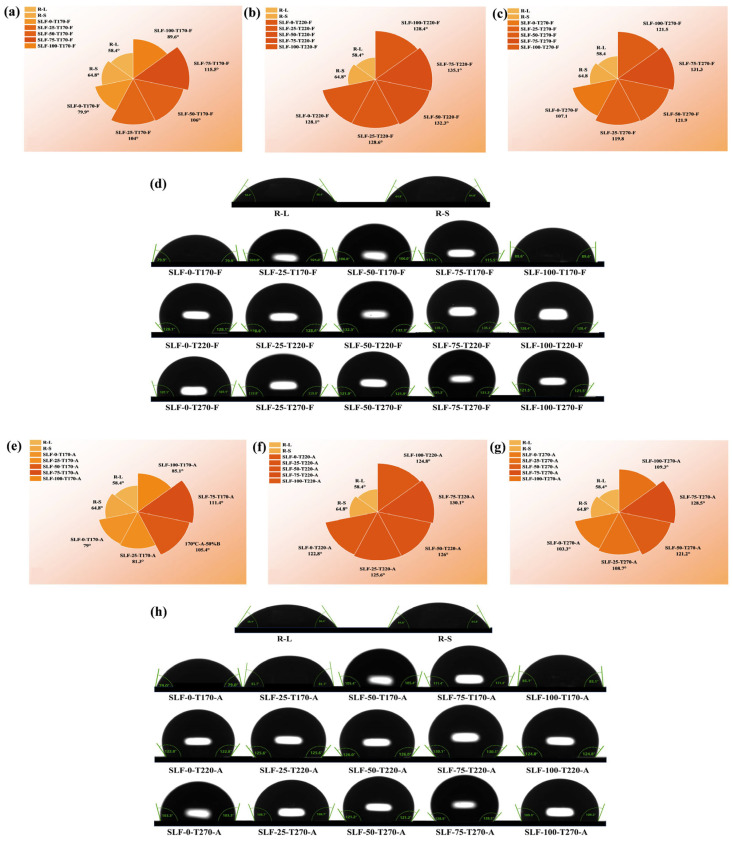
Nightingale diagram of contact angle characteristics; (**a**–**c**) contact angle of upgraded fuel under flue gas atmosphere; (**e**–**g**) contact angle of upgraded fuel under air atmosphere; (**d**,**h**) contact angle of the size of the physical map.

Contact angle measurement serves as a core technique for quantifying coal hydrophobicity [26]. This method involves determining the three-phase contact angle formed by a deionized water droplet at the interface between lignite surfaces and the surrounding air phase [27]. The contact angle value directly correlates with the hydrophilic or hydrophobic nature of coal surfaces. Specifically, a contact angle of θ = 90° indicates a neutral surface, θ < 90° signifies strong hydrophilicity, and θ > 90° demonstrates hydrophobicity. The abundance of hydroxyl (-OH) and carboxyl (-COOH) groups, which tightly bind with water molecules, serves as the key surface chemical characteristic governing hydrophilicity in lignite and wheat straw [28]. During the co-upgradation of lignite (L) and wheat straw (S), hydrophilic functional groups (-OH and -COOH) in cellulose and hemicellulose undergo thermal degradation through pyrolysis, significantly reducing the hydrophilicity of the upgraded SLF fuel. Concurrently, hydrophobic aromatic components derived from pyrolytic tar are deposited on the surfaces of both lignite and wheat straw particles. These aromatic compounds form a hydrophobic coating, enhancing the moisture resistance and hydrophobicity of the SLF fuel. This dual mechanism—decomposition of hydrophilic moieties and surface deposition of hydrophobic aromatics—synergistically improves the fuel’s water-repellent properties, ensuring enhanced storage stability and reduced moisture reabsorption. To assess the hydrophobicity of co-upgraded samples under varying conditions, contact angle measurements were systematically performed on each sample, with detailed results presented in Figure 4. These findings hold significant implications for optimizing upgrading protocols and enhancing the energy applicability of blended fuels.

Obviously, the conditions of the combined refinement pretreatment had a significant effect on the hydrophilicity of the refined blended fuels. Figure 4d and Figure 4h show that R-L and R-S were hydrophilic with contact angles of 58.4° and 64.8°, respectively. The hydrophobicity of the lifted samples was significantly enhanced after the combined lifting pretreatment, as seen in Figure 4a–c,e–g. The hydrophobicity of the SLF fuel was initially enhanced with increasing temperature in the lower temperature range (≤220 °C) during the flue gas atmosphere lifting and drying process and then weakened with increasing temperature up to 270 °C (SLF-0-T220-F > SLF-0-T270-F > SLF-0-T170-F). The trends of the refined samples in an air atmosphere were similar to those in a flue gas atmosphere (SLF-0-T220-A > SLF-0-T270-A > SLF-0-T170-A). The hydrophobicity of the lifted blends obtained under flue a gas atmosphere at 220 °C was overall stronger. The blending of wheat straw also increased its contact angle value, which gradually increased with the increase in the blending ratio of the lifted blended fuels (SLF-75-T220-F > SLF-50-T220-F > SLF-25-T220-F > SLF-100-T220-F > SLF-0-T220-F), and SLF-75-T220-F had a maximum contact angle of 135.1°, exhibiting the strongest hydrophobicity. The hydrophobicity of pure straw extracted fuel (SLF-100-T220-F) was less than that of blended SLF fuel. At 75% straw content, the good compatibility and interfacial interaction with lignite may have facilitated the attachment of hydrophobic aromatic components to the lignite surface and enhanced the hydrophobicity. The lack of some organic matter in the lignite when the straw content was increased to 100% weakened this interaction and led to a decrease in hydrophobicity [17]. Meanwhile, it was found that the contact angle values of the refined blended fuels in an air atmosphere were smaller than those in a flue gas atmosphere. This may be due to the fact that refining in a flue gas atmosphere produces more tars and their hydrophobic aromatic components, which form a hydrophobic layer on the surface of the blended fuels and enhance their hydrophobicity. In contrast, the higher oxygen concentration in air atmosphere promotes further oxidation of these tars, reducing the formation of the hydrophobic layer. The above results show that the blended fuels after joint refining exhibit strong hydrophobicity, which is generally in agreement with the results of the SEM analysis, moisture resorption test, and N_2_ adsorption. Therefore, it can be concluded that the co-extracted samples pretreated with 75% blended wheat straw at a higher temperature (220 °C) in the flue gas atmosphere have stronger hydrophobicity. This treatment not only reduces the operating cost of lignite and biomass drying, but also improves the hydrophobicity of the upgraded blend as well as the efficient utilization of biomass.

#### 2.2.2. Water Reabsorption Experiment

**Figure 5:**
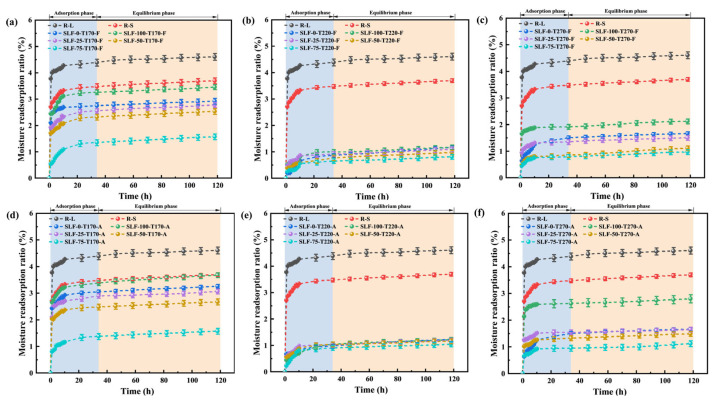
Moisture reabsorption characteristics of upgraded fuels under different conditions at 20 °C, 50% RH: (**a**–**c**): Samples with different mixing ratios from 170 °C to 270 °C in flue gas atmosphere; (**d**–**f**): Samples with different mixing ratios from 170 °C to 270 °C in air atmosphere.

On lignite surfaces, active oxygen-containing functional groups play a pivotal role, with their hydrophilic moieties serving as active sites for moisture reabsorption [29]. The hydrophilic nature of these functional groups provides adsorption sites for atmospheric moisture, effectively facilitating water reabsorption. To comprehensively evaluate the impact of co-upgrading conditions (reaction temperature, blending ratio, reaction atmosphere) on the hydrophilicity of upgraded fuels, moisture reabsorption tests were conducted using a constant temperature and humidity incubator, with results detailed in Figure 5. Based on the moisture reabsorption data of the modified blended fuels prepared by the combined refining process under different conditions, the moisture reabsorption rate (ε) of the feedstock before and after the combined refining process was calculated by Equation (10), which was used as a quantitative index to evaluate its hydrophobicity. The moisture reabsorption phenomenon of lignite involves both physical and chemical adsorption mechanisms. During the first 10 h, the R-L and R-S samples experienced a rapid water reabsorption phase, followed by a significant decrease in the adsorption rate until equilibrium was reached. During the physical adsorption process, water molecules establish temporary intermolecular associations with oxygen-containing groups, including -OH and -COOH groups, on the surface layer of lignite through van der Waals forces and hydrogen bonding. These groups act as adsorption centers, and the higher the content of oxygen groups, the more bridges between adsorbed water molecules, accelerating the adsorption of water [18]. During the chemisorption phase, water molecules form stable chemical bonds with specific functional groups on the surface of lignite. This process is often irreversible and may necessitate the involvement of activation energy. Chemisorption may encompass ion exchange or induce alterations in the chemical structure of the lignite surface, thereby creating a more robust form of water adsorption [30].

**Figure 6:**
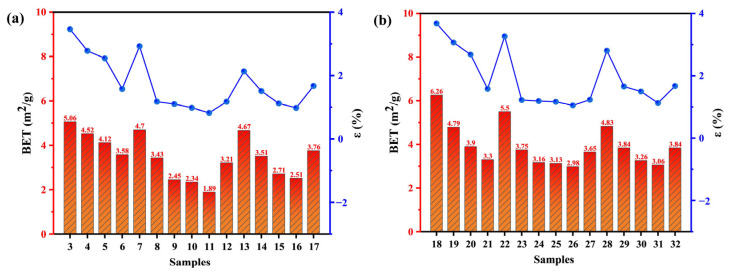
The relationship between ε and BET surface area of different upgraded fuels: (**a**) flue gas atmosphere; (**b**) air atmosphere.

Figure 5a–f demonstrate the hygroscopic curves of upgraded samples under different pretreatment conditions. The SLF fuel subjected to combined upgrading treatment exhibits moisture re-adsorption behaviors analogous to those of R-L and R-S fuels. However, the final equilibrium moisture re-adsorption capacity was significantly reduced. With increasing wheat straw blending ratio, elevated temperature, and variations in reaction atmosphere, the hydrophobicity of modified fuels is significantly enhanced compared to R-L and R-S, with the 75% blending ratio achieving optimal performance (Figure 5a: 1.578%, Figure 5b: 0.821%, Figure 5c: 0.978%, Figure 5d: 1.579%, Figure 5e: 1.051%, Figure 5f: 1.128%). The SLF-75-T220-F fuel exhibits the lowest moisture re-adsorption rate of 0.821%, representing a reduction of 3.790% compared to R-L (4.611%). Figure 6 further presents the experimental results of the combined upgrading treatment fuels in terms of nitrogen adsorption and moisture re-adsorption. It visually demonstrates a direct correlation between hydrophobicity and surface physicochemical structure, particularly porosity and surface chemistry. This relationship arises from moisture re-adsorption dependence on high-energy adsorption sites. Specific surface area (S_BET_) quantifies exposed surface atoms, where greater S_BET_ generally indicates increased abundance of hydrophilic functional groups (–OH, –COOH) and expanded pore structure [18]. The primary adsorption sites exhibit high binding energy and correspond primarily to oxygen-containing functional groups on the coal surface; these sites form hydrogen-bonded monolayers with water molecules. Secondary adsorption sites involve interactions where water molecules adsorb onto previously adsorbed water molecules, leading to multilayer formation. These secondary sites display significantly lower binding energies [31]. Reducing the specific surface area (S_BET_), particularly by decreasing the population of hydrophilic micropores, directly diminishes the availability of high-energy adsorption sites for water molecules, thereby lowering overall hygroscopicity. When investigating the relationship between the pore structure of SLF and its water absorption properties, it can be observed that variations in upgrading temperature markedly impact pore formation. When the temperature is relatively low, the gradual increase in temperature causes the shrinkage effect induced by water evaporation, leading to the collapse of some pore structures on the particle surface. This results in an increase in the number of mesopores and the specific surface area of the particles. The formation of new pores and the expansion of existing pores are thereby promoted, providing numerous pathways for the rapid desorption of water molecules. Once the temperature rises to 270 °C, the combined action of surface contraction forces and cross-linking reactions causes the mesopore structure to begin to disintegrate and gradually close, leading to a decrease in the specific surface area of the particles [30]. In addition to the effects of temperature variations, the addition of wheat straw can enhance the hydrophobic properties of SLF. This enhancement may be attributed to the tar released during the pyrolysis of wheat straw, which forms a hydrophobic film on the surface of lignite. This film significantly strengthens the hydrophobicity of the material. The formation of this film is due to the hydrocarbons present in the tar. These nonpolar molecules have low solubility in water, causing water molecules to arrange themselves in an orderly manner around them. To minimize this ordered arrangement, the nonpolar molecules tend to aggregate, thereby reducing their contact area with water. Moreover, the nonpolar hydrocarbon oils spread on the surface of coal particles, increasing the hydrophobicity of the coal surface and weakening its hydration effect. This makes the coal particles more prone to attaching to gas bubbles and effectively enhances the stability of coal particles’ attachment to bubbles. The adsorption characteristics of tar and its cracking products on the particle surface are closely related to the chemical structure of active functional groups. The -OH groups in phenolic structures promote the formation of non-covalent bonds between molecules through π-π* stacking interactions, thereby constructing a stable multilayer adsorption system. This effect is due to the interaction between the π-electron clouds of phenolic compounds and other aromatic rings, which enhances the intermolecular attraction. Meanwhile, the -COOH structure, with its significant electrostatic properties, can form effective bonds with tar molecules through hydrogen bonding. The oxygen atoms in the carboxyl groups, due to their high electronegativity, attract hydrogen atoms to form hydrogen bonds, further strengthening the intermolecular interactions of tar [32]. These functional groups interact with tar molecules to passivate the hydrophilic groups on the surface of lignite, thereby blocking its pore structure, reducing the contact area with water vapor, and decreasing the moisture adsorption capacity of the lignite surface. The combined action of these physical and chemical changes significantly enhances the hydrophobicity of lignite. However, as the temperature rises to 270 °C, the moisture re-adsorption rate of SLF-75-T270-F increases to 0.978%. This may be because the further increase in temperature during biomass pyrolysis inhibits the adsorption of tar components on the lignite particle surface, making it difficult to form effective adhesion.

### 2.3. Analysis of Combustion Characteristics in Co-Upgraded Raw Materials

#### 2.3.1. Co-Combustion Performance

**Figure 7:**
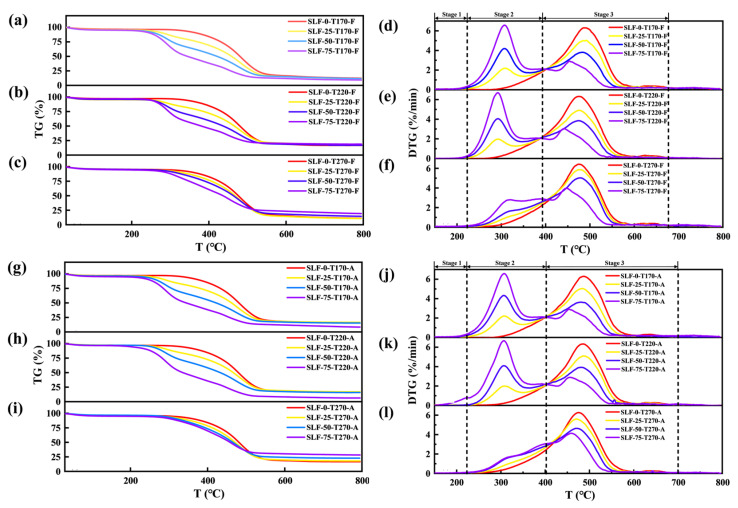
TG curve of co-combustion at a heating rate of 10 °C/min (**a**–**c**) in a flue gas atmosphere and (**g**–**i**) air atmosphere; DTG curve in a (**d**–**f**) flue gas atmosphere and (**j**–**l**) air atmosphere.

Figure 7 presents the TG and DTG combustion characteristic curves of raw materials and blended fuels at a heating rate of 10 °C/min. From the TG curves of co-upgraded samples, the main mass loss stages of blended samples in a flue gas atmosphere occur within 220~670 °C, while those in an air atmosphere span 220~700 °C. For the pretreated SLF fuels, their mass loss is divided into three stages (Stage 1, Stage 2, and Stage 3). The first stage occurs before 220 °C, where the DTG curve of SLF fuel primarily reflects the effective removal of moisture and adsorbed gases. This process is a crucial initial step in combustion. The weight loss during this stage is mainly due to the evaporation of physically adsorbed water and chemically bound water in the fuel, which provides the necessary conditions for subsequent combustion processes. In the temperature range of 220 °C to 400 °C, the second stage is characterized by a broad weight loss peak in the DTG curve. This weight loss is mainly attributed to the substantial release and combustion of volatile matter in the fuel, along with the oxidation of carbon from wheat straw. The release and combustion of volatiles provide the initial heat and reactive substances. As the content of wheat straw increases, the peak combustion rate in the second stage rises continuously, while in the third stage (fixed carbon combustion stage: 400 °C to 700 °C), a downward trend is observed. This change may be due to the depletion of volatile matter, coupled with the relatively slower combustion rate of fixed carbon, leading to an overall decrease in the combustion rate. Additionally, the increase in volatile compounds, the formation of carbonized wheat straw, and the enhanced reduction of lignite may also contribute to this trend [6].

According to Figure 7d–f,j–l, it can be seen that the variation in upgrading temperature has no significant impact on the individual combustion of lignite. For SLF blended fuels, as the temperature increases from 170 °C to 220 °C, the characteristic peaks of the second and third stages shift towards lower temperature regions. This suggests that both the ignition temperature and the burnout temperature exhibit a downward trend. This phenomenon occurs because, within the lower temperature range, the volatile matter content in the mixture is higher than that in coal. At 220 °C, the volatile matter in SLF fuel is effectively released, and its easily ignitable tar cracking products adhere to the surface of the mixture, significantly enhancing the combustion reactivity of the blended sample. However, as the upgrading temperature increases to 270 °C, the peaks gradually shift towards higher temperature regions. This can be attributed to the substantial loss of volatile matter from wheat straw, resulting in a reduced yield of tar cracking products that cannot effectively adhere to the surface of SLF fuel. Additionally, the stability of the macromolecular structure in lignite is enhanced under high-temperature upgrading conditions, while the release of volatile matter and the decomposition of oxygen-containing functional groups are also affected [33]. The TG curves in Figure 7a–c,g–i indicate a reduction in the residual mass of the samples, suggesting that the addition of wheat straw promotes the combustion of lignite. These experimental results are corroborated by the combustion characteristic parameters listed in Table 2. Similar thermodynamic phenomena triggered by the presence of biomass have also been revealed in previous studies [6,34].

#### 2.3.2. Combustion Kinetics

The activation energy represents the energy barrier that molecules must overcome to transition into an activated state capable of undergoing chemical reactions, reflecting the difficulty of initiating the combustion process. During the co-combustion of lignite and wheat straw, SLF-75-T220-F exhibited the lowest activation energy. Compared with other upgraded fuels under different reaction environments, temperatures, and blending ratios, it demonstrated a superior synergistic effect. In this chapter, we applied the KAS and FWO techniques to calculate the activation energies of upgraded fuels under various reaction conditions and blending ratios within the *β* value range of 0.2 to 0.8 for three different heating rates (10, 15, and 20 K/min).

**Figure 8:**
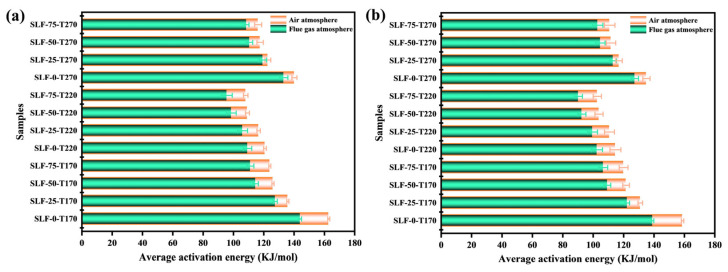
The average value of the activation energy of the combustion process of the mixed fuel under the flue gas atmosphere and the air atmosphere: (**a**) FWO method; (**b**) KAS method.

According to the data in Table 3 and Table 4, the corresponding R^2^ values for the activation energies range from 0.9641 to 0.9999, indicating a high degree of precision in the fitting results. Table 3 and Table 4 provide detailed information on the activation energies and their correlation coefficients (R^2^) for various upgraded fuels under different reaction atmospheres, temperatures, and blending ratios. It can be clearly observed from Table 3 and Table 4 that the differences between the activation energy (*E*_a_) values calculated by the KAS method and the FWO method are minimal across different *β* values. However, the activation energy values obtained by the KAS method are generally lower than those by the FWO method. This discrepancy may arise from differences in the applicability and accuracy of the two methods across different temperature and activation energy ranges, which in turn affect the final results of the activation energy calculations. To minimize computational errors, we adopted the average value of the activation energies obtained by the KAS and FWO methods at different *β* values as the representative activation energy for each sample. As shown in Figure 8a,b, the *E*_a_ values under a flue gas atmosphere are generally lower than those under an air atmosphere. In the simulated flue gas environment, which contains O_2_, H_2_O, and CO_2_, the initial reaction rate of SLF fuel is relatively low. However, with the early occurrence of steam gasification reactions, the reaction rate of char exceeds that under air conditions at lower temperatures. Steam can penetrate a wider range of micropores compared to CO_2_ and O_2_, and it is more conducive to the release of oxygen atoms [35]. The gasification reaction of water vapor may become active at lower temperatures, which helps to reduce the activation energy of the lignite-wheat straw co-upgraded fuel in a flue gas atmosphere. In contrast, in an air atmosphere, the oxidation reaction is more intense due to the higher concentration of oxygen, which may lead to a demand for higher activation energy. When the upgrading temperature is increased from a lower level to 220 °C, the average activation energy of SLF-75-T220-F reaches the lowest value of 95.6 kJ/mol. At 170 °C, the SLF fuel may still be in a slow endothermic stage during the combustion process, requiring higher activation energy. This is because the exothermic effect of chemisorption and the endothermic effect of interfacial reactions coexist in the heat release process, making the chemical reaction the rate-limiting step. In contrast, at upgrading temperatures of 220 °C and 270 °C, the combustion process of SLF fuel may transition to a more rapid endothermic phase, during which adsorption becomes less significant and the reaction is primarily controlled by interfacial reactions, thereby resulting in a relatively lower activation energy. Regardless of the fixed atmosphere or temperature, the activation energy is lowest when the blending ratio of wheat straw is 75%. This indicates that the combustion characteristics of SLF fuel are mainly influenced by the components with higher volatile matter content. This finding is similar to the conclusions drawn by Wang et al. [34]. Based on the above discussion, it can be concluded that the optimal condition for the co-upgradation of lignite and biomass is under a flue gas atmosphere, at 220 °C, with a blending ratio of wheat straw at 75%.

#### 2.3.3. NO Release Analysis

In the nitrogen oxides produced during combustion, nitric oxide (NO) typically dominates, accounting for over 85% of the total, while nitrogen dioxide (NO_2_) is relatively minor, usually within the range of 5–10%. The formation process of NO is highly complex, and its concentration during combustion cannot be easily predicted based solely on the nitrogen content in coal. In this study, we conducted isothermal combustion tests on fuels before and after upgrading to determine NO emissions. By evaluating the efficiency of nitrogen (N) conversion to NO in the fuels, we explored the interactions between wheat straw and lignite and analyzed the effects of upgrading conditions on this conversion efficiency.

**Figure 9:**
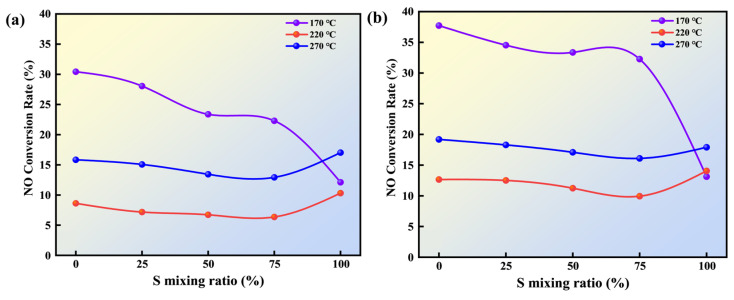
The conversion rate of NO during the combustion process: (**a**) flue gas atmosphere; (**b**) air atmosphere.

Figure 9a and Figure 9b illustrate the conversion rates of N to NO under flue gas and air atmospheres, respectively, at different temperatures and wheat straw blending ratios. As shown in Figure 9, the NO conversion rates under flue gas are generally lower than those under air. According to the elemental analysis in Table 1, the nitrogen content in SLF fuels upgraded under flue gas is almost entirely lower than that in fuels upgraded under air, which fundamentally leads to lower N-to-NO conversion rates in flue gas compared to air. This indicates that the flue gas atmosphere has a significant impact on NO conversion rates. Under flue gas conditions, the NO conversion rate for SLF-0-T170-F is 30.43%, and for SLF-0-T270-F, it is 17.02%. As the wheat straw blending ratio increases, the NO conversion rate of lignite gradually decreases, with the lowest value being 6.36% for SLF-75-T220-F. This demonstrates that the co-upgrading pretreatment of the two fuels reduces the NO conversion rate. The release of NO is primarily influenced by the NO reduction reaction, the intensity of which directly affects the amount of NO released during combustion. In wheat straw fuel, N-5 is the main nitrogen precursor, and studies have shown that N-5 can generate the reducing agent HCN during the volatile release stage [36]. The abundant alkali metal ions in wheat straw catalyze the reduction of HCN, effectively reducing NO formation in lignite and decreasing the conversion of fuel nitrogen to NO. This is the main factor contributing to the reduced NO conversion rate after wheat straw blending.

The upgrading temperature also has a significant impact on the NO conversion rate of SLF fuels. Under a flue gas atmosphere and wheat straw blending conditions, as the upgrading temperature gradually increases to 220 °C, SLF-75-T220-F exhibits the lowest NO conversion rate (6.36%). This may be due to the fact that at higher temperatures within the cryogenic zone range, the proportion of lignite is relatively low, and the tar generated from the pyrolysis of wheat straw sufficiently encapsulates the surface of the lignite in SLF fuel. At the beginning of combustion, a temperature difference forms between the interior and exterior of the particles. Since the ignition temperature of the pyrolyzed tar is high, it forms a tar shell on the surface of the lignite, blocking the pores between lignite particles and reducing the intensity of combustion within the particle interior. As the reaction continues, the fixed carbon in lignite begins to burn, and the tar shell on the lignite surface transforms into pyrolysis products of aromatics with a higher ignition temperature. As a result, a large number of nitrogen-containing compounds are unable to come into contact with oxygen to form NO. When the temperature rises to 270 °C, the NO conversion rate of blended fuels at any blending ratio increases. This may be due to the fact that at higher temperatures, a significant amount of volatile matter is lost from wheat straw during the upgrading process, leading to reduced tar production that cannot effectively adhere to the lignite surface to form a barrier layer, thus participating in subsequent combustion reactions.

### 2.4. Reactivity Characteristics of Pre-Oxidized Semi-Coke from Co-Upgraded Raw Materials

The kinetic analysis of the combustion reaction of the co-upgraded samples obtained at 220 °C in a flue gas atmosphere was found to be optimal, exhibiting the lowest NO conversion rate, as revealed by the NO release analysis in Section 2.3.3. Subsequently, the co-upgraded feedstocks (SLF-0-T220-F, SLF-75-T220-F, and SLF-100-T220-F) under these conditions were subjected to pre-oxidation semi-coke reactions. The combustion reactivity of the semi-coke products and the evolution characteristics of structural nitrogen within the condensed aromatic structures were meticulously analyzed using Raman spectroscopy (Horiba Jobin Yvon, Lab RAM HR800, Longjumeau, France) and X-ray photoelectron spectroscopy (XPS) (Thermo Fisher Scientific Inc., Waltham, MA, USA), respectively. The conversion rates of fixed carbon in each reaction process were determined using Equation (1) [37].

(1) $$R_{c}=1\;-\frac{E_{PP}{\;\times \;A}_{RC}}{E_{RC}{\;\times \;A}_{PA}}$$

where *R_C_* is the conversion rate of fixed carbon; *E_PA_* is the content of carbon element in the pre-treated oxidized semi-coke; *A_RC_* is the ash content in the original lignite; *E_RC_* is the content of carbon element in the original lignite; and *A_PA_* is the ash content in the pre-treated oxidized semi-coke.

#### 2.4.1. Raman Analysis

The microstructural evolution characteristics play a crucial role in influencing the combustion reactivity of coal and biomass fuels [38]. In the experiments, the Raman spectra were precisely fitted using ten typical Gaussian bands, revealing the microstructural changes in the pre-oxidized semi-coke from co-upgraded blended fuels with different mixing ratios under the same temperature and reaction atmosphere [36,39,40]. During the pre-oxidation process in flue gas, which primarily involves reactions with H_2_O and O_2_, lignite particles release a significant number of combustible components, resulting in remarkable changes in the distribution of aromatic ring structures within the semi-coke particles.

**Figure 10:**
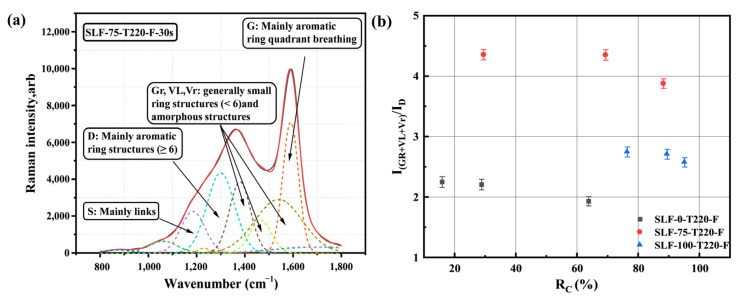
Raman results of sample flue gas pretreatment of oxidized semi-coke; (**a**) Raman spectrum deconvolution example; (**b**) Correlation between I _(Gr+VL+Vr)_/I_D_ values and *R*_C_.

As shown in Figure 10b, the relationship between the Raman band area ratio I_(Gr+VL+Vr)_/I_D_ and the conversion rate *R*_C_ of the semi-coke from three co-upgraded samples is illustrated. For the same sample, as the reaction time increases (30–60–90 s), the conversion rate *R*_C_ gradually rises, indicating an increase in the consumption of fixed carbon. This is attributed to the prolonged contact and reaction time between the fixed carbon and air. In the initial stage, the surface reaction of the fixed carbon is relatively rapid. However, as time progresses, the reaction gradually penetrates into the interior of the carbon particles, potentially leading to an optimized state of combustion reaction kinetics. Compared with the individually upgraded fuels SLF-0-T220-F and SLF-100-T220-F, when the biomass blending ratio is increased to 75%, the value of I_(Gr+VL+Vr)_/I_D_ increases under the same reaction time conditions (30 s, 60 s, or 90 s). This trend can be attributed to the enhanced structural evolution of the semi-coke with an increased biomass blending ratio, which promotes the aromaticity and structural stability of the carbonaceous material. The primary reasons for the observed phenomena are as follows: During the co-combustion process of lignite and biomass, the alkali metal components in lignite can accelerate electron transfer, thereby promoting the chemisorption of oxygen atoms at active sites to form oxygen-containing functional groups. This process enhances the combustion reactivity of the fuel. This synergistic effect enhances the combustion reaction efficiency, accelerating the consumption of carbon atoms in large aromatic structures and promoting the formation of small sized aromatic ring structures. Previous studies have also indicated that water vapor exerts a significant influence on the polymerization process of aromatic hydrocarbon structures within semi-coke particles [37]. In simulated flue gas, the presence of 10% water vapor promotes the gasification reaction between semi-coke particles and water molecules, accelerating the destruction of aromatic ring structures and leading to the formation of defect structures and the release of carbonaceous gaseous products. Moreover, the substantial oxidative hydroxyl radicals (•OH) generated from the decomposition of water molecules can activate large aromatic ring systems with relatively low reactivity, thereby promoting the activation and cleavage of these structures into smaller aromatic ring structures [41]. The pre-oxidation process conducted under oxygen-deficient conditions produces a large number of •OH, which in turn leads to the formation of a significant number of tar cracking products (small sized aromatic hydrocarbon components) during the pyrolysis of wheat straw. These products tend to adhere to the surface of SLF fuel, thereby altering the surface characteristics of the aromatic hydrocarbon components of the fuel. During the pre-oxidation process, active sites or defect structures on the particle surface adsorb oxygen atoms to form oxygen-containing functional groups. These functional groups are more likely to be desorbed through the cleavage of C-C bonds, thereby generating small-sized aromatic ring structures, long-chain aliphatic compounds, and gaseous products [42]. Therefore, it can be inferred that at higher temperatures (220 °C), the oxidant content in the flue gas atmosphere can effectively promote the depolymerization of condensed aromatic structures into smaller aromatic ring structures. Moreover, a high proportion of wheat straw blending (75%) can further intensify this phenomenon.

#### 2.4.2. XPS Analysis

**Figure 11:**
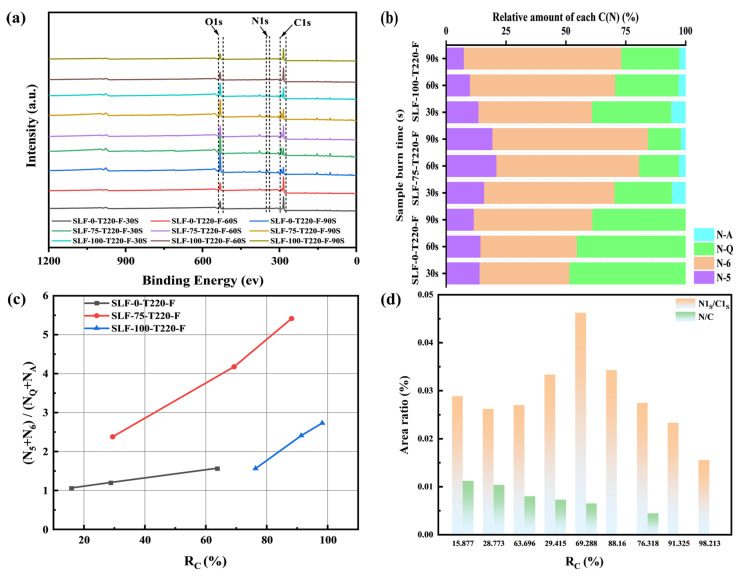
XPS results of sample flue gas pretreatment of oxidized semi-coke; (**a**) XPS full spectrum; (**b**) area percentage of N-A, N-Q, N-5, N-6 XPS; (**c**) distribution of nitrogenous functional groups; (**d**) ratio variations in N1S/C1S to N/C.

In the study of surface elements in lignite, biomass, and co-upgraded blended fuels, XPS is frequently employed to analyze the differences in the chemical states of surface elements. As shown in Figure 11a, the XPS full spectra of SLF-0-T220-F, SLF-75-T220-F, and SLF-100-T220-F are presented. The characteristic peaks at binding energies of 284 eV and 398 eV correspond to C1s and N1s, respectively. The ratio of N1s/C1s in the XPS data can reflect the relative content of nitrogen and carbon elements on the surface of the coke particles, thereby providing a reference for analyzing the changes in the samples during the reaction process. As shown in Figure 11d, the variations in N1s/C1s and N/C ratios are presented. For SLF-75-T220-F, the N1s/C1s ratio is consistently higher than that of SLF-0-T220-F and SLF-100-T220-F under any degree of combustion. This phenomenon may be attributed to the transformation of amino groups into aromatic nitrogen compounds or the formation of stable C-N bonds with carbon atoms. These reactions enable nitrogen atoms to exist in a more stable chemical form, leading to the enrichment of nitrogen on the particle surface. The surface of the upgraded blended fuel is coated with a substantial number of tar pyrolysis products (small polycyclic aromatic hydrocarbons). These small polycyclic aromatic hydrocarbons react with O_2_, wherein the aromatic ring structures capture oxygen molecules via π-π interactions, thereby forming peroxy radicals (ROO•). Subsequently, peroxy radicals are transformed into more stable peroxides or other radical intermediates through hydrogen atom transfer or addition reactions. These intermediates further decompose or react with other radicals to generate a series of oxygen-containing products while releasing additional radicals that propagate the reaction chain [43,44]. These chain reactions consume oxygen, reducing the opportunities for nitrogen elements within the fuel to come into contact with oxygen, thereby decreasing the probability of NO formation.

Nitrogen-containing functional groups are generally regarded as precursors to nitrogenous gaseous products, and they determine the emission characteristics of structural nitrogen during various reaction processes [45]. Therefore, it can be inferred that the distribution of nitrogen-containing functional groups significantly influences the emission pathways of residual nitrogen in the semi-coke generated after each co-upgrading pre-oxidation process during subsequent combustion. Typically, four types of nitrogen-containing functional groups are present on the surface of coal-based fuels, namely N-A (amide nitrogen), N-5 (pyrrolic nitrogen), N-6 (pyridinic nitrogen), and N-Q (quaternary nitrogen). N-A is typically present in biomass and rapidly disappears during combustion, having no significant impact on the experimental results; hence, it is not analyzed in this study [46]. N-5 is typically located at the periphery of aromatic ring structures. Due to its relatively low C-N bond energy, it is prone to cleavage during the pyrolysis process. This bond cleavage reaction leads to the transformation of N-5 into gaseous nitrogen-containing products, such as HCN and NH_3_. N-Q is typically located within polycyclic aromatic structures or graphite layers and exhibits high thermal stability. Owing to its structural stability, N-Q is less likely to decompose under high-temperature conditions and is thus considered a precursor to NO formation [47]. Lignite has a relatively low degree of metamorphism, with a more disordered carbon structure. However, after pre-treatment at elevated temperatures and durations, the degree of graphitization can be significantly enhanced. Figure 11b illustrates the percentage distribution of nitrogen-containing functional groups on the surface of the blended fuel. The results indicate that N-Q is the predominant form of structural nitrogen in the pre-oxidized semi-coke of SLF-0-T220-F.

To determine the impact of biomass blending ratios on the evolution of nitrogen-containing functional groups during the flue gas pre-oxidation process, the results shown in Figure 11b,c indicate that, under constant reaction temperature and atmosphere, increasing the wheat straw blending ratio (0 vol%→75 vol%) positively promotes the formation and enrichment of N-5 and N-6 on the surface of pre-oxidized semi-coke particles, at the expense of N-Q. The abundant alkali metal elements in biomass play a significant role in the evolution of surface structures. This evolution, in turn, has a noticeable impact on the distribution of nitrogen atoms, leading to a shift in nitrogen-containing functional groups from N-Q to N-5 and N-6. The presence of a certain amount of O_2_ in flue gas may activate aromatic ring structures. This activation accelerates the transformation of carbon atoms from initially stable and less reactive condensed aromatic structures to gaseous and defective structures. The desorption of carbon atoms weakens the thermal stability of condensed structures, leading to the disintegration of large aromatic systems and promoting the migration of some internal nitrogen atoms (mainly N-Q) towards the structural periphery. At higher temperatures (220 °C), the presence of water vapor in flue gas facilitates the penetration of small hydrogen molecules into the matrix of semi-coke particles. This process significantly enhances the selective consumption of smaller aromatic ring structures and cross-linked structures within anthracite particles, as well as condensation reactions. Therefore, during the flue gas pre-oxidation process, nitrogen atoms originally located at the periphery of certain aromatic structures may shift to internal nitrogen-containing structures. The primary pathway for the evolution of structural nitrogen is the transformation of N-Q into N-5, at the expense of N-Q. Fuel pretreatment enables source-level mitigation of NO_x_ precursors during combustion, directly reducing operational demands on downstream denitrification systems. This approach enhances cost efficiency in coal-fired power plants by minimizing catalyst attrition and reagent consumption. Concurrently, suppressed NO_x_ emissions curtail atmospheric nitrate aerosol formation, thereby alleviating secondary PM2.5 pollution and benefiting ecological nitrogen cycle equilibrium. To optimize the distribution of nitrogen-containing functional groups on the surface of semi-coke and promote their conversion into precursors of HCN and NH_3_, it is recommended, based on the results of this study, to conduct the pre-treatment under a flue gas atmosphere at a temperature of 220 °C, with a lignite proportion of 25% and a wheat straw proportion of 75%.

## 3. Experimental

### 3.1. Sample Preparation

**Figure 12:**
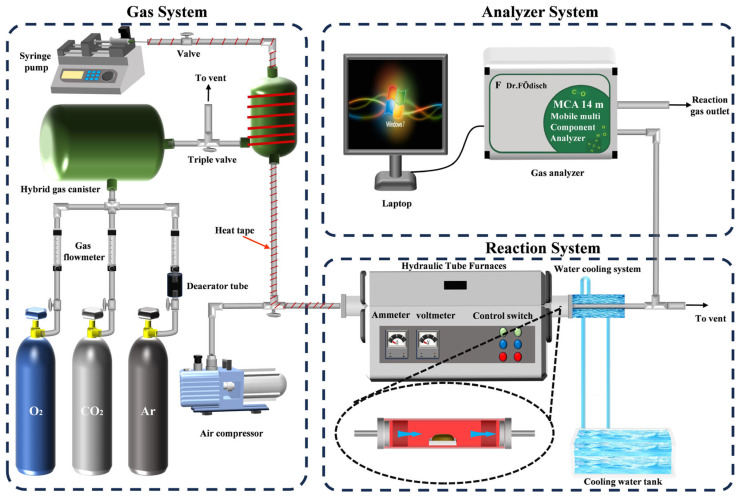
Experimental system of lignite and wheat straw combined upgrading.

Lignite (R-L) from Inner Mongolia Autonomous Region, China, and wheat straw (R-S) from Heilongjiang Province, China, were used as raw materials. Prior to experiments, the lignite and wheat straw samples were pulverized and sieved to achieve a uniform particle size distribution (90–125 μm), and blended fuels with wheat straw mass ratios of 0%, 25%, 50%, 75%, and 100% were prepared. The low-temperature co-upgrading pretreatment was conducted using a fixed-bed reactor system (Figure 12), comprising a gas distribution system, reactor, and flue gas analysis subsystem, though only the first two subsystems were utilized in this study. For each experiment, 0.4 ± 0.003 g of blended fuel was precisely loaded into the reactor. A reactive gas flow of 2 L/min was maintained in a left-to-right direction through the sample bed, significantly enhancing gas–solid contact efficiency while suppressing secondary reactions caused by particle stacking, thereby minimizing experimental interference [48]. Upon entering the reaction zone, lignite particles rapidly underwent heat and mass exchange with the hot gas stream: surface moisture evaporation absorbed latent heat, lowering particle temperature, which subsequently promoted the condensation of pyrolysis-derived tar cracking products from wheat straw onto the cooled lignite surfaces.

The low-temperature co-upgrading pretreatment was conducted at 170 °C, 220 °C, and 270 °C to explore the effect of different temperatures on the in situ migration of hydrophobic components. Based on prior optimization studies, the pretreatment duration was fixed at 30 min [49]. Reactive atmospheres included air and simulated flue gas (6% O_2_ + 10% CO_2_ + 10% H_2_O + 74% Ar) to replicate industrial boiler emission conditions. Post treatment, samples were rapidly cooled to ambient temperature using an ice-water bath quenching system, collected, and subjected to comprehensive characterization to evaluate pretreatment efficacy. Detailed sample nomenclature is provided in the nomenclature table.

### 3.2. Elemental Analysis and Industrial Analysis

Elemental composition (C, H, N, S) was determined using an elemental analyzer (vario MACRO cube, Anhalt, Germany). Proximate analysis (ash and volatile matter content) was conducted via the combustion method in a muffle furnace [50]. The calorific value was calculated according to Equation (2) [51].

(2) HHV=0.3491C+1.1783H+0.1005S −0.1034O −0.0151N −0.0211A

### 3.3. Combustion Characteristic Determination

Combustion characteristics of raw materials and co-upgraded wheatstraw–lignite blended fuels were investigated using a thermogravimetric analyzer (SDT Q600, TA Instruments, New Castle, DE, USA). Experiments were performed under an air atmosphere with a flow rate of 100 mL/min. Approximately 5 mg of co-upgraded sample was loaded into an alumina crucible and heated from 30 °C to 800 °C at a heating rate of 10 °C/min. Under dynamic conditions, samples were monitored for mass loss (TG curves) and rate of mass loss (DTG curves), which demonstrate the mass dynamics of co-upgraded samples over time and temperature. Ignition temperature (T_a_) and burnout temperature (T_b_) were determined via the tangent method [52].

### 3.4. Kinetic Analysis

In the kinetic analysis of combustion reactions, we derived temperature-dependent intrinsic reaction rate constants through the integration of electronic structure theory and application of statistical rate theory. The accuracy of these rate constants relies not only on the precision of electronic structure theory in characterizing potential energy surfaces and single-point energy calculations but also on the validity of the employed statistical rate theory. The evolution of char conversion efficiency during combustion can be characterized by two distinct functions: a temperature-dependent function *k*(*T*) and a conversion-dependent function *f*(β). Based on the Arrhenius equation and kinetic relationships, the combustion conversion rate was calculated using Equation (3):

(3) $$\frac{d\beta }{\mathrm{dt}}\;=\;k(T)f(\beta )\;=\;Aexp\left(-\frac{E_{a}}{RT}\right)f(\beta )$$

where *t* represents time, *T* denotes reaction temperature, A is the pre-exponential factor, *E_a_* signifies activation energy, *f*(β) corresponds to the reaction mechanism function, and *R* is the universal gas constant (R *R*= 8.314 J/(mol·K^−1^)).

During combustion, the conversion ratio β serves as a metric quantifying the extent of sample conversion, defined by Equation (4):

(4) $$\beta \;=\;\frac{m_{0}\;-m_{t}}{m_{0}\;-m_{h}}$$

where *m*_0_ and *m_h_* represent the initial mass and final mass of the sample, respectively, while *m_t_* denotes the mass of the sample at time *t*.

The function *f(*β*)* is defined as:

(5) $$f(\beta )\;=\;{\left(1\;-\beta \right)}^{n}$$

Given that the reaction temperature *T* can be expressed as a function of the heating rate (*γ*) and time (*t*), Equation (3) is reformulated as:

(6) $$\frac{\;d\beta }{\mathrm{dT}}\;=\;\frac{A}{\gamma }\exp\left(-\frac{E_{a}}{\mathrm{RT}}\right){\left(1\;-\beta \right)}^{n}$$

g(β) is expressed as the integral form of *f*(β), and after integration, Equation (6) is rewritten as Equation (7):

(7) $$g(\beta )=\int_{0}^{\beta }\frac{d\beta }{f\left(\beta \right)}=\frac{A}{\gamma }\int_{T_{0}}^{T}\exp\left(-\frac{E_{a}}{\mathrm{RT}}\right)\mathrm{dT}$$

where *T* = *T*_0_ + *γt*.

Under conditions of uncertain reaction mechanism functions, isoconversional methods enable accurate estimation of combustion activation energy by relying solely on temperature-dependent reaction rates. Two model-free approaches—the Kissinger–Akahira-Sunose (KAS) method and Flynn–Wall–Ozawa (FWO) model—were employed to calculate kinetic parameters [34,53]. The governing equations are given by Equations (8) and (9):

(8) $$\ln\left[\frac{g(\beta )}{T^{2}}\right]\;=\;\ln\left(\frac{AR}{\gamma E}\right)-\frac{E_{a}}{\mathrm{RT}}$$

(9) $$\ln[g(\beta )]=\ln\left[\frac{{AE}_{a}}{R\beta }\right]-5.331\;-1.052\frac{E_{a}}{\mathrm{RT}}$$

To ascertain kinetic parameters, non-isothermal thermogravimetric experiments were performed on 10 mg samples at varying heating rates (10, 15, and 20 K/min). Activation energy was calculated via the linear plot method. Under the same conditions and procedures, three repeated experiments were conducted, and the average value was taken.

### 3.5. Physical Property Characterization

#### 3.5.1. Pore Structure Characterization

The effects of low-temperature co-upgrading pretreatment on the porous structure of blended fuels were evaluated using a nitrogen adsorption analyzer (ASAP 2020M, Micromeritics Instrument Corp., Norcross, GA, USA). Measurements were conducted at −196 °C. The specific surface area was determined via the Brunner–Emmett–Teller (BET) method, while pore size distribution was calculated using the Barrett–Joyner–Halenda (BJH) model [54,55].

#### 3.5.2. Topography Characterization

The morphological evolution of co-upgraded blended fuels was characterized using a Hitachi SU-8000 scanning electron microscope (SEM) (Hitachi, Tokyo, Japan). Images were captured at an accelerating voltage of 10 kV. To enhance image clarity, all samples were sputter-coated with a gold film prior to analysis. Distinct morphological alterations on lignite particle surfaces were visually confirmed, directly evidencing the in situ migration and deposition of aromatic components derived from biomass pyrolysis.

### 3.6. Isothermal Combustion Experiment

To investigate the isothermal combustion characteristics of co-upgraded blended fuels, combustion tests were conducted in the fixed-bed experimental setup (as shown in Figure 12). A sample mass of 0.3 ± 0.003 g was reacted with air at a flow rate of 1.5 L/min under isothermal conditions (800 °C). Gaseous NO emissions were monitored in real time using a flue gas analyzer (VA MCA 14 m, Dr. Födisch Umweltmesstechnik AG, Hangzhou, China). The conversion rate of structural nitrogen to NO gaseous products was calculated using Equation (10) [37].

(10) $$\eta_{\mathrm{NO}}=\frac{1000\;\times \;M_{N}\;\times \;\frac{Q}{60\times 22.4}\;\times \;\int_{0}^{t}(C_{\mathrm{NO}}\;\times \;10^{-6})\mathrm{dt}}{m\;\times \;f_{N}}$$

where *η*_NO_ is the conversion rate of NO gaseous products, %; 0 is the start time of the reaction, that is, the start of the integral; t is the end time of the reaction, that is, the end point of the integral; dt is the integration variable; *M*_N_ is the molar mass of nitrogen, g/mol; *Q* is the flow rate of the reaction gas, L/min; *C*_NO_ is the volume fraction of NO; m is the mass of the low-temperature upgraded sample, mg; and *f*_N_ is the percentage of nitrogen in the upgraded sample, %.

Upon reaching the target reactor temperature of 800 °C, co-upgraded samples were rapidly introduced into the reaction zone. By controlling residence times (30 s, 60 s, and 90 s), semi-coke samples with varying combustion degrees were generated to investigate nitrogen evolution. These semi-coke samples were analyzed through elemental analysis and X-ray photoelectron spectroscopy (XPS) to elucidate the migration mechanisms of structural nitrogen within condensed aromatic frameworks and their correlation with low-temperature co-upgrading pretreatment conditions. The combined approach enables precise tracking of nitrogen redistribution from fuel matrices to aromatic systems under controlled thermal exposure, providing critical insights into nitrogen retention and transformation pathways during staged combustion.

### 3.7. Co-Upgraded Fuel Semi-Coke Raman Spectroscopy Analysis

In this study, a micro-Raman spectrometer (Horiba Jobin Yvon, Lab RAM HR800, Longjumeau, France) equipped with an Nd-YAG laser (532 nm) was used to determine the microscopic structural characteristics of the semi-coke samples after co-upgradation and combustion. An appropriate laser wavelength was selected as the excitation source, the laser output power was adjusted to 5 MW, and the laser beam was precisely focused on the surface of the target semi-coke particles by scanning on the sample surface. The Raman spectra covered a wavenumber range of 100~4000 cm^−1^. Within the Raman spectral region of 800~1800 cm^−1^, ten Gaussian peaks were deconvoluted [39]. Specific Raman spectral bands, namely G, G_r_, V_L_, V_r_, D, and S, were used to reveal the microscopic structural characteristics of coal-based fuels [40]. Among them, the G band (~1580 cm^−1^) originates from the E_2g_ symmetric vibration of sp^2^ carbon atoms, and the D band (~1350 cm^−1^) originates from the A_1g_ symmetric vibration of sp^2^ carbon atoms [56].

### 3.8. Co-Upgraded Fuel Semi-Coke XPS Analysis

In this study, a Thermo Scientific Nexsa G2 X-ray Photoelectron Spectrometer (Thermo Fisher Scientific Inc., Waltham, MA, USA) was employed, which is capable of providing high-quality surface analysis results. This facilitates the elucidation of the evolution characteristics of nitrogen-containing functional groups on the surface of semi-coke samples from blended fuels under different co-upgrading pretreatment conditions. Prior to testing, the samples were thoroughly dried for 24 h in a vacuum desiccator at a temperature of 105~110 °C to ensure that physically adsorbed gas molecules on the particle surfaces were fully removed. During the analysis, all obtained electron spectra were calibrated and corrected using the C1s peak (284.8 eV), a step crucial for ensuring the accuracy of the spectra. To determine the specific distribution of various nitrogen-containing functional groups on the surface of pre-oxidized semi-coke, charge calibration, peak identification, and peak fitting were performed using Avantage 6.9 software (Thermo Fisher Scientific Inc., Waltham, MA, USA). In the Raman spectra of N1s, there are four characteristic peaks, with their specific energy positions as follows: N-6 at 398.8 ± 0.1 eV, N-5 at 400.4 ± 0.1 eV, N-Q at 401.4 ± 0.1 eV, and the peak position of N-X is within the range of 402.0~404.0 eV [45]. These peaks correspond to different forms of nitrogen. Among them, N-5, N-6, and N-Q are commonly regarded as key precursors for the formation of nitrogen oxides.

### 3.9. Hydrophilicity Analysis

#### 3.9.1. Contact Angle Characterization

The effects of low-temperature co-upgrading pretreatment conditions on the hydrophobicity of blended fuels were determined using a contact angle measuring instrument (OSA200-B, LAUDA Scientific, Lauda-Königshofen, Germany). Prior to testing, 100 mg of the co-upgraded fuel, which had been formed into a sheet, was dried for 24 h at 105~110 °C to eliminate the influence of inherent moisture content [57]. To ensure uniform and comparable surface characteristics across all fuel samples, a standardized pelletization protocol was implemented: precisely weighed aliquots (1000.0 ± 0.5 mg) of dried powder were loaded into cylindrical stainless steel dies (20 mm diameter) and compacted at 10 MPa for 15 s using a hydraulic pellet press (MC, MITR, Changsha, China). The static contact angle on the surface of the coal sample was measured using the sessile drop method with deionized water. Each measurement lasted for 20 s.

#### 3.9.2. Moisture Re-Absorption Test

The hygroscopic characteristics of raw lignite, wheat straw and low-temperature upgraded lignite, and wheat straw and blended fuels were investigated by a moisture reabsorption experiment using a constant humidity and temperature chamber (HSP-250BE, Li Chen, Shanghai, China). The hydrophilicity of each upgraded sample under different upgrading conditions was determined. The relative humidity for the hygroscopic test was 50% RH, and the experimental temperature was 20 °C. Prior to the experiment, the upgraded samples were dried for 24 h at 60 °C in a drying oven and then stored in a sealed container. For each run, approximately 1.3 ± 0.003 g of the sample was evenly placed in a petri dish. During the first 10 h, the sample mass was measured every hour, and after 10 h, the sample mass was measured every 12 h. The moisture reabsorption experiment reached equilibrium when the mass change of the sample between two consecutive measurements under the same reabsorption conditions was less than 0.1%. The ε value of each sample, used to evaluate the hydrophobicity of the samples after co-upgradation, can be calculated using Equation (11) [17]. The effects of storage environment and surface structural characteristics on the moisture reabsorption process of low-temperature co-upgraded blended fuels were determined. Under the same conditions and procedures, three repeated experiments were conducted, and the average value was taken.

(11) $${\varepsilon =(m}_{1}-m_{0}{)/m}_{0}\;\times \;100\%$$

where m_0_ is the weight of the upgraded sample before water adsorption, and m_1_ is the weight of the upgraded sample after water adsorption.

## 4. Conclusions

This study conducts co-upgrading experiments of wheat straw and lignite under different atmospheres, temperatures, and blending ratios and systematically analyzes and characterizes the modified samples. The results indicate that the co-upgrading effect of the blended fuel is optimal under the conditions of a flue gas atmosphere, 220 °C, and a wheat straw blending ratio of 75%. However, for industrial-scale deployment, variations in flue gas composition necessitate additional purification, while inherent density differences between coal and biomass impede uniform mixing under continuous feeding conditions. Furthermore, disparities in biomass composition and coal origin significantly influence tar yield and adsorption performance; this study specifically utilized Inner Mongolia lignite and Heilongjiang wheat straw. Consequently, the hydrophobicity and combustibility of upgraded fuels derived from high-ash lignite or potassium-rich biomass require further investigation. The specific conclusions are summarized as follows:

(1) Under flue gas conditions, the calorific value of the co-upgraded samples exhibits significant enhancement. When the reaction temperature reaches 220 °C, the most pronounced improvement in fuel quality is observed, effectively enhancing the combustion reactivity of the modified fuel. However, further temperature elevation results in substantial depletion of combustible components with radical activity, consequently diminishing the combustion reactivity of the modified fuel.
(2) Samples subjected to low-temperature treatments and high-ratio blending exhibit a diminished specific surface area and demonstrate a smoother surface morphology, thereby significantly weakening the moisture re-adsorption capacity of the modified fuels. Through the combined action of physical restructuring and chemical transformations, the hydrophobicity of the modified fuels achieves remarkable enhancement.
(3) High-ratio biomass blending facilitates the in situ migration and adsorption of organic constituents onto lignite particle surfaces. These organic components undergo pyrolytic decomposition during combustion to generate reductive gaseous products, which actively participate in the homogeneous reduction reaction of NO, thereby effectively suppressing NO formation during the combustion process.

## Figures and Tables

**Table 1:** Proximate and ultimate identification of lignite, straw, and upgraded fuel.

Samples	Ultimate Analysis (daf) (wt.%)				Proximate Analysis (ar) (wt.%)				HHV (MJ/kg)
	C	H	N	O	Mar	Ash	Volatile	FC	
1. R-L	56.23	4.69	1.48	37.60	6.84	17.64	17.32	58.20	23.65
2. R-S	35.75	5.56	2.04	56.64	7.49	14.38	42.78	35.35	17.40
3. SLF-0-T170-F	67.00	3.97	0.87	28.16	3.56	16.77	17.84	61.83	26.72
4. SLF-25-T170-F	65.50	3.76	0.83	29.92	3.66	15.84	36.45	44.05	25.95
5. SLF-50-T170-F	64.84	3.63	0.81	30.73	3.27	15.44	40.46	40.83	25.57
6. SLF-75-T170-F	64.24	3.42	0.76	31.58	2.52	14.99	42.23	40.26	25.13
7. SLF-100-T170-F	50.87	6.93	0.99	41.21	3.46	20.67	35.41	40.46	24.39
8. SLF-0-T220-F	77.94	3.63	0.77	17.67	3.83	18.18	17.78	60.21	30.19
9. SLF-25-T220-F	75.59	2.99	0.74	20.67	5.70	18.77	25.25	50.28	28.59
10. SLF-50-T220-F	74.00	2.65	0.75	22.60	5.17	19.98	23.25	51.60	27.59
11. SLF-75-T220-F	71.93	2.48	0.72	24.87	5.14	20.48	16.99	57.39	26.63
12. SLF-100-T220-F	53.91	6.49	0.47	39.14	4.17	21.49	29.45	44.89	24.98
13. SLF-0-T270-F	62.27	3.15	0.62	33.96	4.65	13.26	18.33	63.76	24.15
14. SLF-25-T270-F	62.36	2.75	0.53	34.36	4.99	13.99	27.07	53.95	23.70
15. SLF-50-T270-F	64.17	2.94	0.49	32.41	4.89	17.66	22.69	54.76	24.49
16. SLF-75-T270-F	66.04	2.68	0.42	30.86	5.79	20.67	18.35	55.19	24.79
17. SLF-100-T270-F	53.00	6.80	0.51	39.70	4.57	23.73	24.41	47.29	24.97
18. SLF-0-T170-A	62.87	6.00	1.53	29.61	2.94	20.96	17.61	58.49	27.51
19. SLF-25-T170-A	60.61	5.55	1.40	32.44	3.38	19.58	40.99	36.05	26.22
20. SLF-50-T170-A	59.23	5.39	1.32	34.07	2.93	18.35	42.11	36.61	25.56
21. SLF-75-T170-A	53.25	4.61	1.17	40.97	2.37	10.06	45.56	42.01	22.69
22. SLF-100-T170-A	44.48	6.89	0.87	47.76	2.66	21.11	32.16	44.07	22.07
23. SLF-0-T220-A	61.49	4.58	1.21	32.72	2.97	16.84	18.15	62.04	25.44
24. SLF-25-T220-A	60.46	4.42	1.16	33.96	3.10	16.07	41.57	39.26	24.91
25. SLF-50-T220-A	59.02	4.22	1.09	35.67	3.03	15.13	42.56	39.28	24.18
26. SLF-75-T220-A	54.00	3.77	0.98	41.25	4.01	7.76	48.09	40.14	22.02
27. SLF-100-T220-A	54.74	4.04	0.45	40.77	3.41	22.90	27.81	45.88	22.33
28. SLF-0-T270-A	55.72	5.18	1.08	38.02	3.86	17.63	17.43	61.08	24.09
29. SLF-25-T270-A	57.21	4.99	1.03	36.78	4.28	19.11	23.16	53.45	24.37
30. SLF-50-T270-A	60.13	4.98	1.05	33.85	5.43	23.77	29.18	41.62	25.31
31. SLF-75-T270-A	60.74	4.71	1.01	33.55	5.20	25.24	31.30	38.26	25.17
32. SLF-100-T270-A	58.39	2.53	0.46	38.63	3.77	24.32	22.16	49.75	21.80

daf = dry-ash-free ar = air dry basis, daf = dry-ash-free ar = air dry basis. Mar: Moisture; Ash: Ash content; Volatile: Volatile matter; FC: Fixed carbon; HHV: Higher heating value.

**Table 2:** Combustion characteristic parameters of samples before and after combined refining.

Samples	T_a_ (°C)	T_b_ (°C)	DTG_a_ (%/min)	DTG_m_ (%/min)	Samples	T_a_ (°C)	T_b_ (°C)	DTG_a_ (%/min)	DTG_m_ (%/min)
SLF-0-T170-F	396.54	647.88	1.14	9.90	SLF-0-T170-A	395.28	734.11	1.09	6.31
SLF-25-T170-F	366.67	638.00	1.15	5.13	SLF-25-T170-A	249.59	732.95	1.08	5.05
SLF-50-T170-F	246.21	641.34	1.15	4.21	SLF-50-T170-A	248.61	726.92	1.09	4.33
SLF-75-T170-F	250.87	630.29	1.19	6.59	SLF-75-T170-A	254.55	719.68	1.06	8.84
SLF-0-T220-F	400.14	644.20	1.07	6.33	SLF-0-T220-A	400.22	656.27	1.08	6.33
SLF-25-T220-F	250.83	639.39	1.05	4.91	SLF-25-T220-A	251.87	651.66	1.06	5.12
SLF-50-T220-F	247.19	636.33	1.07	4.05	SLF-50-T220-A	250.87	650.34	1.08	4.09
SLF-75-T220-F	244.89	634.85	1.05	6.70	SLF-75-T220-A	245.59	643.57	1.20	6.67
SLF-0-T270-F	400.18	647.64	1.16	6.43	SLF-0-T270-A	401.52	729.97	1.07	6.27
SLF-25-T270-F	382.90	645.95	1.16	5.87	SLF-25-T270-A	386.58	726.28	1.05	5.61
SLF-50-T270-F	365.37	641.31	1.11	5.04	SLF-50-T270-A	371.65	722.69	1.00	4.64
SLF-75-T270-F	294.37	643.59	1.05	3.98	SLF-75-T270-A	343.94	718.12	0.92	4.17

**Table 3:** Activation energy of mixed fuel (SLF) was obtained by KAS method and FWO method under flue gas atmosphere, different reaction temperatures, and fuel blending ratios.

Conversion Ratio *β*	FWO		KAS		Conversion Ratio β	FWO		KAS	
	E_a_ (kJ/mol)	R^2^	E_a_ (kJ/mol)	R^2^		E_a_ (kJ/mol)	R^2^	E_a_ (kJ/mol)	R^2^
**SLF-0-T170-F**					**SLF-25-T170-F**				
0.2	154.2	0.9957	150.7	0.9950	0.2	128.3	0.9999	124.9	0.9999
0.3	147.0	0.9990	142.7	0.9988	0.3	126.4	0.9989	121.9	0.9988
0.4	145.8	0.9983	141.1	0.9979	0.4	126.3	0.9963	121.2	0.9956
0.5	143.7	0.9978	138.6	0.9973	0.5	128.8	0.9774	123.3	0.9730
0.6	140.9	0.9978	135.4	0.9972	0.6	127.7	0.9930	121.9	0.9916
0.7	138.4	0.9971	132.5	0.9964	0.7	127.4	0.9962	121.2	0.9955
0.8	138.7	0.9978	132.6	0.9972	0.8	128.6	0.9979	122.2	0.9974
Average	144.1		139.1		Average	127.6		122.4	
**SLF-50-T170-F**					**SLF-75-T170-F**				
0.2	118.3	0.9705	115.0	0.9657	0.2	114.1	0.9844	110.9	0.9817
0.3	113.8	0.9980	109.9	0.9976	0.3	111.0	0.9875	107.3	0.9851
0.4	106.7	0.9985	101.6	0.9982	0.4	105.7	0.9999	101.5	0.9999
0.5	109.7	0.9733	104.0	0.9672	0.5	108.6	0.9972	104.1	0.9967
0.6	119.0	0.9736	113.2	0.9677	0.6	114.3	0.9911	109.4	0.9893
0.7	118.2	0.9903	111.9	0.9879	0.7	113.7	0.9937	108.1	0.9923
0.8	116.2	0.9852	109.5	0.9815	0.8	111.1	0.9943	104.6	0.9928
Average	114.6		109.3		Average	111.2		106.6	
**SLF-0-T220-F**					**SLF-25-T220-F**				
0.2	107.7	0.9960	101.8	0.9951	0.2	106.2	0.9810	101.5	0.9772
0.3	109.3	0.9999	103.0	0.9999	0.3	101.3	0.9899	95.5	0.9876
0.4	110.1	0.9982	103.5	0.9977	0.4	95.4	0.9658	88.8	0.9569
0.5	109.8	0.9952	103.0	0.9938	0.5	116.3	0.9641	110.3	0.9563
0.6	108.5	0.9933	101.4	0.9913	0.6	107.3	0.9967	100.3	0.9960
0.7	108.4	0.9926	101.1	0.9904	0.7	108.9	0.9986	101.6	0.9981
0.8	111.5	0.9945	104.0	0.9929	0.8	106.7	0.9793	98.7	0.9736
Average	109.3		102.5		Average	106.0		99.5	
**SLF-50-T220-F**					**SLF-75-T220-F**				
0.2	95.6	0.9823	91.1	0.9786	0.2	98.4	0.9989	94.4	0.9987
0.3	97.3	0.9815	92.4	0.9775	0.3	94.7	0.9950	90.2	0.9940
0.4	103.5	0.9813	98.1	0.9773	0.4	96.6	0.9985	91.8	0.9982
0.5	96.3	0.9869	89.9	0.9837	0.5	95.7	0.9729	90.5	0.9669
0.6	101.7	0.9980	95.1	0.9975	0.6	97.0	0.9673	91.3	0.9596
0.7	107.5	0.9623	100.8	0.9532	0.7	94.5	0.9753	87.9	0.9688
0.8	88.2	0.9654	79.9	0.9535	0.8	92.5	0.9914	85.2	0.9890
Average	98.6		92.5		Average	95.6		90.2	
**SLF-0-T270-F**					**SLF-25-T270-F**				
0.2	134.0	0.9994	129.6	0.9993	0.2	126.9	0.9960	122.5	0.9953
0.3	137.0	0.9783	132.3	0.9744	0.3	118.4	0.9991	112.9	0.9990
0.4	133.1	0.9951	127.8	0.9941	0.4	112.0	0.9999	105.7	0.9999
0.5	138.7	0.9774	133.3	0.9729	0.5	118.5	0.9667	112.2	0.9590
0.6	125.5	0.9879	119.0	0.9851	0.6	99.2	0.9841	91.2	0.9791
0.7	138.0	0.9901	131.6	0.9879	0.7	129.1	0.9718	122.2	0.9652
0.8	125.9	0.9732	118.5	0.9665	0.8	132.0	0.9879	124.8	0.9849
Average	133.2		127.4		Average	119.4		113.1	
**SLF-50-T270-F**					**SLF-75-T270-F**				
0.2	102.0	0.9878	97.1	0.9852	0.2	111.2	0.9838	107.1	0.9808
0.3	91.8	0.9727	86.0	0.9655	0.3	104.0	0.9850	99.1	0.9818
0.4	113.7	0.9756	108.5	0.9703	0.4	107.3	0.9957	102.0	0.9948
0.5	110.0	0.9939	104.3	0.9924	0.5	105.9	0.9823	100.0	0.9783
0.6	107.5	0.9979	100.8	0.9972	0.6	106.2	0.9733	99.9	0.9671
0.7	123.5	0.9968	117.2	0.9959	0.7	112.6	0.9947	106.2	0.9933
0.8	125.8	0.9676	119.2	0.9605	0.8	113.0	0.9784	106.1	0.9729
Average	110.6		104.7		Average	108.6		102.9	

**Table 4:** Activation energy of mixed fuel (SLF) was obtained by KAS method and FWO method under air atmosphere, different reaction temperatures, and fuel blending ratios.

Conversion Ratio *β*	FWO		KAS		Conversion Ratio *β*	FWO		KAS	
	*E*_a_ (kJ/mol)	R^2^	*E*_a_ (kJ/mol)	R^2^		*E*_a_ (kJ/mol)	R^2^	*E*_a_ (kJ/mol)	R^2^
**SLF-0-T170-A**					**SLF-25-T170-A**				
0.2	155.8	0.9825	152.4	0.9798	0.2	131.6	0.9844	128.4	0.9998
0.3	178.6	0.9893	176.0	0.9879	0.3	142.9	0.9853	139.3	0.9819
0.4	169.5	0.9787	166.1	0.9756	0.4	136.1	0.9992	131.5	0.9828
0.5	149.1	0.9742	144.3	0.9697	0.5	146.8	0.9882	142.4	0.9991
0.6	172.5	0.9706	168.6	0.9660	0.6	131.2	0.9998	125.6	0.9862
0.7	150.7	0.9746	145.3	0.9698	0.7	125.2	0.9990	118.8	0.9998
0.8	164.1	0.9892	159.0	0.9872	0.8	137.3	0.9932	131.0	0.9875
Average	162.9		158.8		Average	135.9		131.0	
**SLF-50-T170-A**					**SLF-75-T170-A**				
0.2	126.8	0.9773	124.0	0.9739	0.2	134.5	0.9982	132.2	0.9980
0.3	123.4	0.9921	120.1	0.9908	0.3	120.4	0.9920	117.1	0.9906
0.4	127.4	0.9931	123.7	0.9919	0.4	117.2	0.9731	113.4	0.9684
0.5	122.9	0.9829	118.2	0.9797	0.5	110.7	0.9954	106.1	0.9945
0.6	127.1	0.9875	122.0	0.9851	0.6	122.3	0.9919	117.8	0.9904
0.7	125.2	0.9853	119.6	0.9823	0.7	134.2	0.9734	129.7	0.9685
0.8	129.7	0.9920	123.8	0.9904	0.8	128.9	0.9867	123.7	0.9842
Average	126.1		121.6		Average	124.0		120.0	
**SLF-0-T220-A**					**SLF-25-T220-A**				
0.2	125.2	0.9875	120.3	0.9849	0.2	108.3	0.9868	103.7	0.9840
0.3	124.2	0.9787	118.7	0.9742	0.3	116.5	0.9905	111.4	0.9885
0.4	113.6	0.9882	107.2	0.9853	0.4	104.4	0.9879	98.18	0.9847
0.5	115.7	0.9997	109.1	0.9996	0.5	123.0	0.9743	117.3	0.9687
0.6	127.3	0.9907	121.1	0.9886	0.6	122.8	0.9893	116.8	0.9868
0.7	120.0	0.9866	113.2	0.9833	0.7	119.3	0.9892	112.8	0.9866
0.8	120.0	0.9680	112.9	0.9602	0.8	121.5	0.9934	114.8	0.9917
Average	120.8		114.6		Average	116.6		110.7	
**SLF-50-T220-A**					**SLF-75-T220-A**				
0.2	106.2	0.9878	102.2	0.9855	0.2	108.0	0.9770	104.6	0.9729
0.3	95.0	0.9933	90.2	0.9919	0.3	95.8	0.9980	91.0	0.9976
0.4	105.6	0.9999	100.6	0.9999	0.4	100.2	0.9643	95.3	0.9564
0.5	109.9	0.9995	104.4	0.9993	0.5	113.2	0.9987	108.5	0.9984
0.6	110.9	0.9897	104.8	0.9872	0.6	115.6	0.9783	110.4	0.9737
0.7	121.2	0.9897	115.4	0.9875	0.7	112.8	0.9834	107.0	0.9797
0.8	115.5	0.9716	108.9	0.9706	0.8	111.4	0.9678	105.0	0.9603
Average	109.2		103.8		Average	108.1		102.7	
**SLF-0-T270-A**					**SLF-25-T270-A**				
0.2	126.0	0.9994	121.1	0.9992	0.2	123.8	0.9983	119.3	0.9980
0.3	135.0	0.9999	130.2	0.9999	0.3	115.0	0.9930	109.4	0.9915
0.4	149.1	0.9783	144.7	0.9744	0.4	130.5	0.9960	125.3	0.9953
0.5	165.9	0.9672	162.0	0.9620	0.5	120.9	0.9900	114.9	0.9879
0.6	134.0	0.9958	128.2	0.9948	0.6	122.0	0.9898	115.8	0.9875
0.7	130.9	0.9958	124.8	0.9948	0.7	122.4	0.9929	116.0	0.9913
0.8	140.3	0.9958	134.3	0.9872	0.8	124.6	0.9950	118.0	0.9940
Average	140.2		135.0		Average	122.7		117.0	
**SLF-50-T270-A**					**SLF-75-T270-A**				
0.2	114.2	0.9827	109.6	0.9794	0.2	113.9	0.9904	109.6	0.9886
0.3	106.7	0.9731	101.1	0.9672	0.3	104.0	0.9916	98.7	0.9897
0.4	126.3	0.9705	121.3	0.9649	0.4	117.0	0.9989	111.9	0.9986
0.5	143.2	0.9707	138.7	0.9657	0.5	116.1	0.9722	110.6	0.9665
0.6	116.2	0.9827	110.0	0.9788	0.6	127.1	0.9824	121.7	0.9789
0.7	100.5	0.9780	92.9	0.9714	0.7	114.2	0.9936	107.8	0.9919
0.8	116.0	0.9872	108.6	0.9837	0.8	122.1	0.9785	115.4	0.9734
Average	117.6		111.7		Average	116.3		110.8	

## Data Availability

Data are contained within the article.

## Nomenclature

R-L	Untreated lignite
R-S	Untreated straw
SLF-0-T170-F	Upgraded fuel (straw 0%) obtained from flue gas atmosphere at 170 °C
SLF-25-T170-F	Upgraded fuel (straw 25%) obtained from flue gas atmosphere at 170 °C
SLF-50-T170-F	Upgraded fuel (straw 50%) obtained from flue gas atmosphere at 170 °C
SLF-75-T170-F	Upgraded fuel (straw 75%) obtained from flue gas atmosphere at 170 °C
SLF-100-T170-F	Upgraded fuel (straw 100%) obtained from flue gas atmosphere at 170 °C
SLF-0-T220-F	Upgraded fuel (straw 0%) obtained from flue gas atmosphere at 220 °C
SLF-25-T220-F	Upgraded fuel (straw 25%) obtained from flue gas atmosphere at 220 °C
SLF-50-T220-F	Upgraded fuel (straw 50%) obtained from flue gas atmosphere at 220 °C
SLF-75-T220-F	Upgraded fuel (straw 75%) obtained from flue gas atmosphere at 220 °C
SLF-100-T220-F	Upgraded fuel (straw 100%) obtained from flue gas atmosphere at 220 °C
SLF-0-T270-F	Upgraded fuel (straw 0%) obtained from flue gas atmosphere at 270 °C
SLF-25-T270-F	Upgraded fuel (straw 25%) obtained from flue gas atmosphere at 270 °C
SLF-50-T270-F	Upgraded fuel (straw 50%) obtained from flue gas atmosphere at 270 °C
SLF-75-T270-F	Upgraded fuel (straw 75%) obtained from flue gas atmosphere at 270 °C
SLF-100-T270-F	Upgraded fuel (straw 100%) obtained from flue gas atmosphere at 270 °C
SLF-0-T170-A	Upgraded fuel (straw 0%) obtained from air gas atmosphere at 170 °C
SLF-25-T170-A	Upgraded fuel (straw 25%) obtained from air gas atmosphere at 170 °C
SLF-50-T170-A	Upgraded fuel (straw 50%) obtained from air gas atmosphere at 170 °C
SLF-75-T170-A	Upgraded fuel (straw 75%) obtained from air gas atmosphere at 170 °C
SLF-100-T170-A	Upgraded fuel (straw 100%) obtained from air gas atmosphere at 170 °C
SLF-0-T220-A	Upgraded fuel (straw 0%) obtained from air gas atmosphere at 220 °C
SLF-25-T220-A	Upgraded fuel (straw 25%) obtained from air gas atmosphere at 220 °C
SLF-50-T220-A	Upgraded fuel (straw 50%) obtained from air gas atmosphere at 220 °C
SLF-75-T220-A	Upgraded fuel (straw 75%) obtained from air gas atmosphere at 220 °C
SLF-100-T220-A	Upgraded fuel (straw 100%) obtained from air gas atmosphere at 220 °C
SLF-0-T270-A	Upgraded fuel (straw 0%) obtained from air gas atmosphere at 270 °C
SLF-25-T270-A	Upgraded fuel (straw 25%) obtained from air gas atmosphere at 270 °C
SLF-50-T270-A	Upgraded fuel (straw 50%) obtained from air gas atmosphere at 270 °C
SLF-75-T270-A	Upgraded fuel (straw 75%) obtained from air gas atmosphere at 270 °C
SLF-100-T270-A	Upgraded fuel (straw 100%) obtained from air gas atmosphere at 270 °C
XPS	X-ray photoelectron spectroscopy
Raman	Raman spectra
R_C_	Conversion rate of carbon content after pretreatment
ε	Amount of water reabsorption
